# Roles of MOV10 in Animal RNA Virus Infection

**DOI:** 10.3389/fvets.2020.569737

**Published:** 2020-09-16

**Authors:** Feng Su, Xueming Liu, Yunliang Jiang

**Affiliations:** Shandong Provincial Key Laboratory of Animal Biotechnology and Disease Control and Prevention, College of Animal Science and Veterinary Medicine, Shandong Agricultural University, Tai'an, China

**Keywords:** animal RNA virus, antivirus mechanism, MOV10, miRNA pathways, mRNA

## Abstract

Animal epidemic diseases caused by RNA viruses are the primary threat to the livestock industry, and understanding the mechanisms of RNA virus clearance from target cells is critical to establish an effective method to reduce economic losses. As an SF-1, ATP-dependent RNA helicase in the UPF1p family, MOV10 participates in the RNA degradation of multiple viruses mediated via miRNA pathways and therefore contributes to a decrease in the replication of RNA viruses. This review primarily focuses on the bioactivity of MOV10, the mechanism of RNA virus removal, and the potential roles of MOV10 in RNA virus clearance. In addition, clues are provided to reduce animal diseases caused by RNA viruses.

Animal epidemic diseases caused by RNA viruses are the primary threat to the livestock industry (1), and understanding the mechanisms of RNA virus clearance in target cells can lead to an effective method to reduce economic losses. Therefore, this review focuses on the current understanding of the mechanisms of viral resistance to RNA viruses that cause animal diseases.

## Diseases Caused by RNA Viruses in the Livestock Industry

Animal epidemic diseases are the primary threat to the livestock industry. Of the veterinary pathogens that cause these diseases, viruses are responsible for approximately half of the most important animal diseases, according to the OIE's (Office of International Epizootic) classification of terrestrial and aquatic notifiable animal diseases (1). Animal viruses are divided into DNA and RNA viruses on the basis of the genetic materials, both the viruses are the dominate pathogens that affecting animal production in livestock industry (1, 2). The differences between DNA and RNA viruses include their lifetimes in target cells, how they attach to and enter host cells, and their biosynthesis, maturation, and release from cells (2, 3).

Compared with DNA viruses, RNA viruses have a higher mutation rate and cause more serious economic damage in the livestock industry (4). Lower mutation rates of DNA viruses were usually influence by the viral genome and DNA repairing protein that benefit for proofread and correct replication errors (5). Oppositely, offspring of RNA viruses are usually produced 1 ± 2 mutations compared with their parent. RNA virus usually producing a mutant cloud of descendants in extremely environment (5). RNA viruses are also divided into avian, mammalian, and zoonotic viruses in domestic animal industries. For example, the avian leukosis retrovirus causes great losses in the avian industry and many subgroups have been isolated in recent years (6–12). In mammals, more RNA viruses are also being reported, of which the classical swine fever virus and porcine reproductive and respiratory syndrome virus (PRRSV) are the main RNA viruses that cause great losses in porcine industries. Because of their high mutation rates and the additional subgroups identified in recent years (13–17), both viruses are difficult to eliminate. Some RNA viruses not only infect animals but also humans, with the influenza virus the most typical RNA zoonotic virus. For example, H5N1 and H9N2 subgroups of the influenza virus are the main infectious pathogens affecting both humans and animals (18–23). To reduce economic losses in animal industries, the main approaches are to reduce the virus titer in animals and to improve their immunity. Thus, an understanding of the mechanism that clears RNA viruses from target cells is the most important step in controlling virus infection.

## MIRNA Pathways Are Important in the Clearance of Animal RNA Viruses

**Figure 1:**
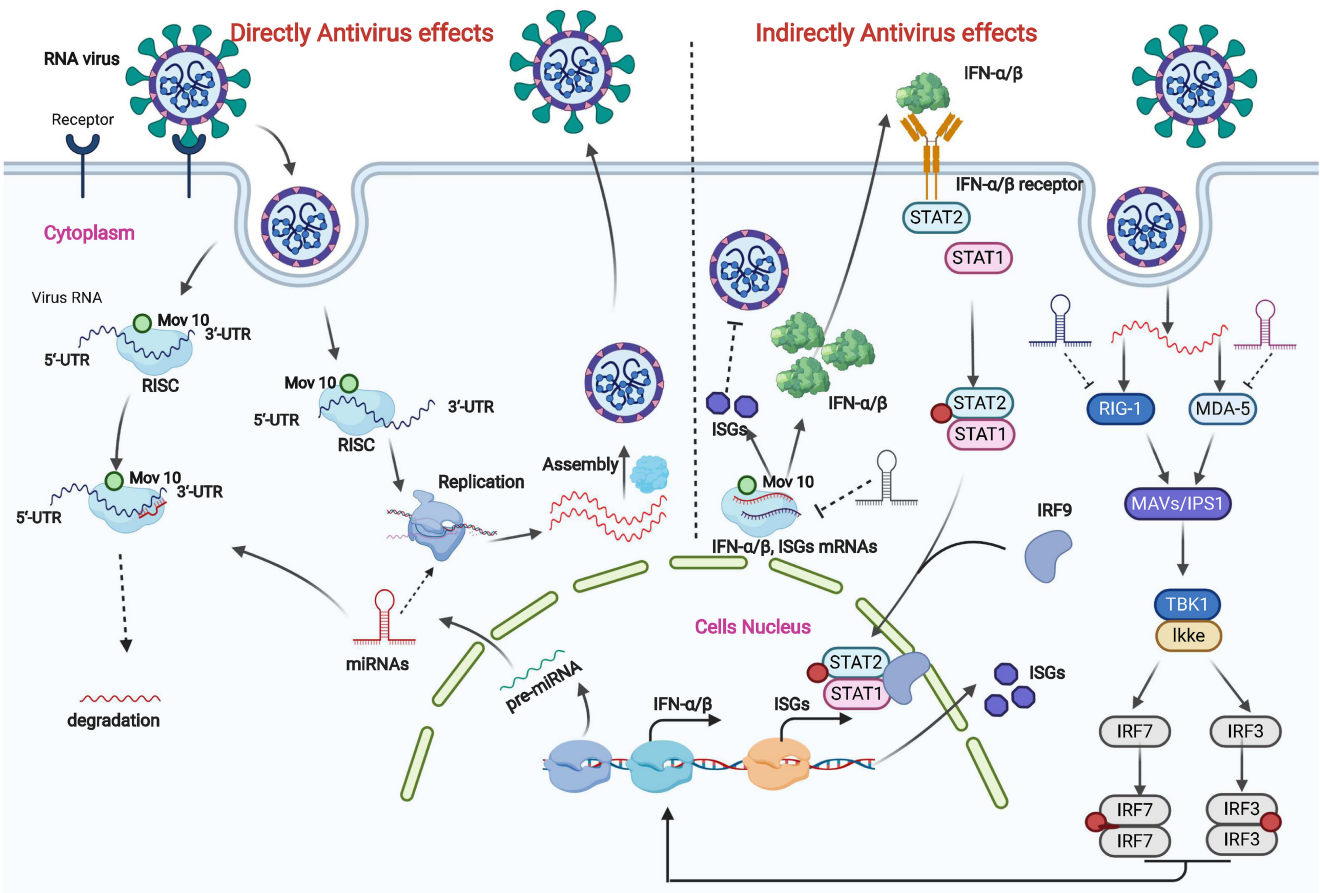
Direct and indirect antivirus effects of miRNAs in target cells. Antivirus effects of miRNAs are divided into direct and indirect regulation in target cells. In direct regulation, host miRNAs participate in viral RNA degradation in a RISC within a P body in target cells and are also involved in viral RNA replication processes by targeting RNA replication components. Indirect antiviral effects are complex. First, host miRNAs participate in the RIG-1 and MDA-5 pathway-mediated IFN-α/β production. The production of IFN-α/β not only inhibits viral replication in cells but also activates the IFN signal pathway and ultimately causes the expression of IFN-stimulated genes (ISGs) to decrease virus replication. miRNAs also participate in this regulation process.

RNA interference (RNAi) is an important mechanism in mediating the clearance of RNA viruses (24). Viral RNA can be degraded through the inhibition of viral replication by the binding effects between small interfering RNAs and viral nucleic acids (24, 25). Generally, the antiviral effects due to RNAi are typically found in invertebrates, and RNAi functioning as an antiviral effector has only been detected in undifferentiated stem cells (26, 27). RNAi also plays important roles in the activation of IFN-I (Type I interferon) and its antiviral effectors (IFN-stimulated genes), with those genes producing the main antiviral effects in differentiated vertebrate somatic cells (Figure 1) (28).

RNA virus replication and clearance are typically influenced by several factors, of which miRNAs produced in cells may play important roles in a regulated network. miRNAs usually bind to the miRNA binding sites within mRNAs and viral genomes to exert their regulatory function (29, 30). The number of miRNA binding sites within a viral genome affects the viral replication (Figure 1). Most miRNA binding sites are in the 5' and 3' non-translation regions (NTRs) of a viral genome (31), but the sites were also recently found in the translated regions (32, 33). An increase in the number of perfect complementary miRNA binding sites in the NTRs of RNA viruses causes strong translational repression *in vitro* and a decrease in virulence in mice (34). miRNA gene translation is also influenced by epigenetic and environmental conditions (34, 35). For example, compared with primary tissues, low levels of miRNAs are detected in cultural cells, especially in a high confluency (36). Furthermore, non-canonical miRNA target sites in specific cells are also potential factors that affect miRNA production and therefore RNA virus replication (25).

Direct inhibition effects of miRNAs on viruses were previous recognized as an effective way in invertebrates rather than in vertebrates, subsequent studies confirmed the anti-viral function of miRNAs in undifferential vertebrates' stem cells. But the direct viral inhibition effect of miRNAs was controversial in somatic Cells (37). Cullen's studies evaluated the directly viral genome targeting effects of miRNAs and his research revealed viruses induced miRNAs are hardly causing reduction of virus copy number (38–40). Recently study summarized the potential directly viral genome targeting effects of miRNAs. Trobaugh's review analyzed the direct inhibitory effects of miRNAs. And the review point out that direct inhibitory effects of miRNAs on viruses are primarily concentrated in the viral genome and in viral genome translation (Figure 1) (31, 41). There are so many reasons causing direct targeting reduction of miRNAs, of which viruses escaping mechanism, incomplete pairing of miRNA and viruses mutation are the important reasons. But the artificial application of miRNAs on viruses genome were reported as an important method to reduce viral replication copies recently.

Direct inhibitory effects of miRNAs on viruses mainly play its roles in viral genome and in viral genome translation. Positive-strand RNA viruses release their genomes into the cytoplasm after entering cells following recognition by receptors within the membrane surface (41). Similar to mRNA produced in a cell, the released viral RNA is either decayed or replicated when combined with miRNAs in the cytoplasm. For example, the expression of miR-181, miR-206, miR-23, and miR-378 in specific cells can inhibit the replication of PRRSV by binding to the viral genome (42, 43). By contrast, miR-17 and let-7c bind to the 3' NTR of the bovine viral diarrhea virus genome and cause virus genome translation and an increase in RNA stability rather than translational repression (41). Similar effects are also found in negative-strand viruses. The negative-stand RNA released in the cytoplasm is first translated into positive-stand RNA for protein synthesis and virus assembly. The translated positive RNA can recruit miRNA binding to the miRNA-recognized sites and ultimately influence virus replication and assembly (25). For example, the influenza PB1 gene recruits miR-323, miR-491, miR-485, miR-654, and miR-3145, which leads to RNA degradation in infected human and canine epithelial cell lines *in vitro* (33, 44, 45).

To determine the indirect effects of miRNAs on virus infection, the primary focus is on the level of miRNAs and the changes in host immunity response in target cells (Figure 1) (46). Specifically, an immune response initiated by a virus mediates viral-recognition receptors that lead to activation of translation factors and ultimately changes in the expression profiles of miRNAs (47, 48). For example, changes in the expression of specific miRNAs (miR-151-5p and miR-223-3p) determine whether the H5N2 influenza virus is pathogenic or is attenuated (49). Changes in specific miRNAs also affect miRNA-mediated protein expression (50). The IFN systems are a crucial mechanism in the removal of viral infection (25). Virus-mediated regulation of the IFN signaling cascade is controlled by miRNA levels in target cells (25). For example, miR-23 and miR-505 can increase IFN-α/β expression, which is useful in clearing PRRSV (42, 43). By contrast, with the expression of miR-23b, avian leukosis viral infection in chicken spleen downregulates IFN regulatory factor 1 levels and innate immune induction (51). Thus, the miRNAs that are involved in downregulating the innate immune response during RNA virus infections need to be identified in the future.

Though the indirect effects of miRNAs on RNA viral infection were widely confirmed and recognized, the direct effects of miRNAs were still controversial. This controversy was mainly concentrated on several factors. Firstly, miRNAs were reducible generated from the cells after the viruses infection, but these miRNAs were hardly presented its' directly target effects on viral genome because of the low level of miRNAs produced by the target cells. The other factor is the potential escaping mechanism of viral and the cells complex immune system. The mismatch of miRNAs and viral genome mutation also reduced its inhibition effects.

## MOV10 Is an Important Regulator of Animal RNA Virus Invasion

MOV10 is an important SF-1, ATP-dependent RNA helicase in the UPF1p family (52–55), originally identified as a protein that prevents Moloney murine leukemia virus infection in mice. Two potential mechanisms can explain the antiviral bioactivity of MOV10: (1) regulating antiviral gene expression so as to achieve antiviral capacity (56) and (2) changing miRNA expression directly or indirectly so as to mediate viral clearance effects (46). The two mechanisms are discussed below in detail.

### MOV10 Regulates Antiviral Gene Expression by Different Pathways

The antiviral activity of MOV10 has been evaluated and detected in many studies. The infectivity of different viruses is regulated by the MOV10 helicase, for example, human hepatitis delta virus (57), human immunodeficiency virus type 1 (HIV-1), and dengue virus (58). The same effect is also observed with PRRSV infection (59). Different genes and pathways are activated in specific virus-infected target cells. In HIV-infected cells, the antiviral activity of MOV10 is detected in multiple stages in original studies. MOV10 is efficiently incorporated into virions and reduces virus infectivity by inhibiting reverse transcription in the first stage (52). Overexpression of APOBEC3G and MOV10 reduces proteolytic processing of HIV-1 Gag, which effectively reduces HIV replication in the later stage (60). Recent studies show that MOV10 inhibits the degradation of APOBEC3G through interfering with the Vif-mediated (Vif: HIV-1-encoded virion infectivity factor protein) ubiquitin–proteasome pathway (61–63). MOV10 can also provide antiviral activity against RNA viruses by increasing the induction of RIG-I–MAVS-independent IFN (56) and through IKKϵ and IRF3, the induction of IFN (Figure 1) (64). In influenza A virus-infected models, MOV10 with nucleoprotein (NP) promotes viral RNA degradation through the lysosomal pathway (65). In addition, MOV10 sequesters the ribonucleoprotein (RNP) of influenza A virus in the cytoplasm and is antagonized by viral influenza non-structural (NS1) protein (66).

In a recent study of MOV10 that focused on animal disease, MOV10 directly inhibited replication of the PRRSV by retaining the viral nucleocapsid protein in the cytoplasm of Marc-145 cells (59). MOV10 also inhibits replication of the murine leukemia virus (67). Many genes potentially activated by MOV10 exhibit strong antiviral bioactivity (58). IRAV (FLJ11286) is an interferon-stimulated gene with antiviral activity against dengue virus that interacts with MOV10 (58).

### MOV10 Mediates Inhibition of RNA Viruses via miRNA Pathways

**Figure 2:**
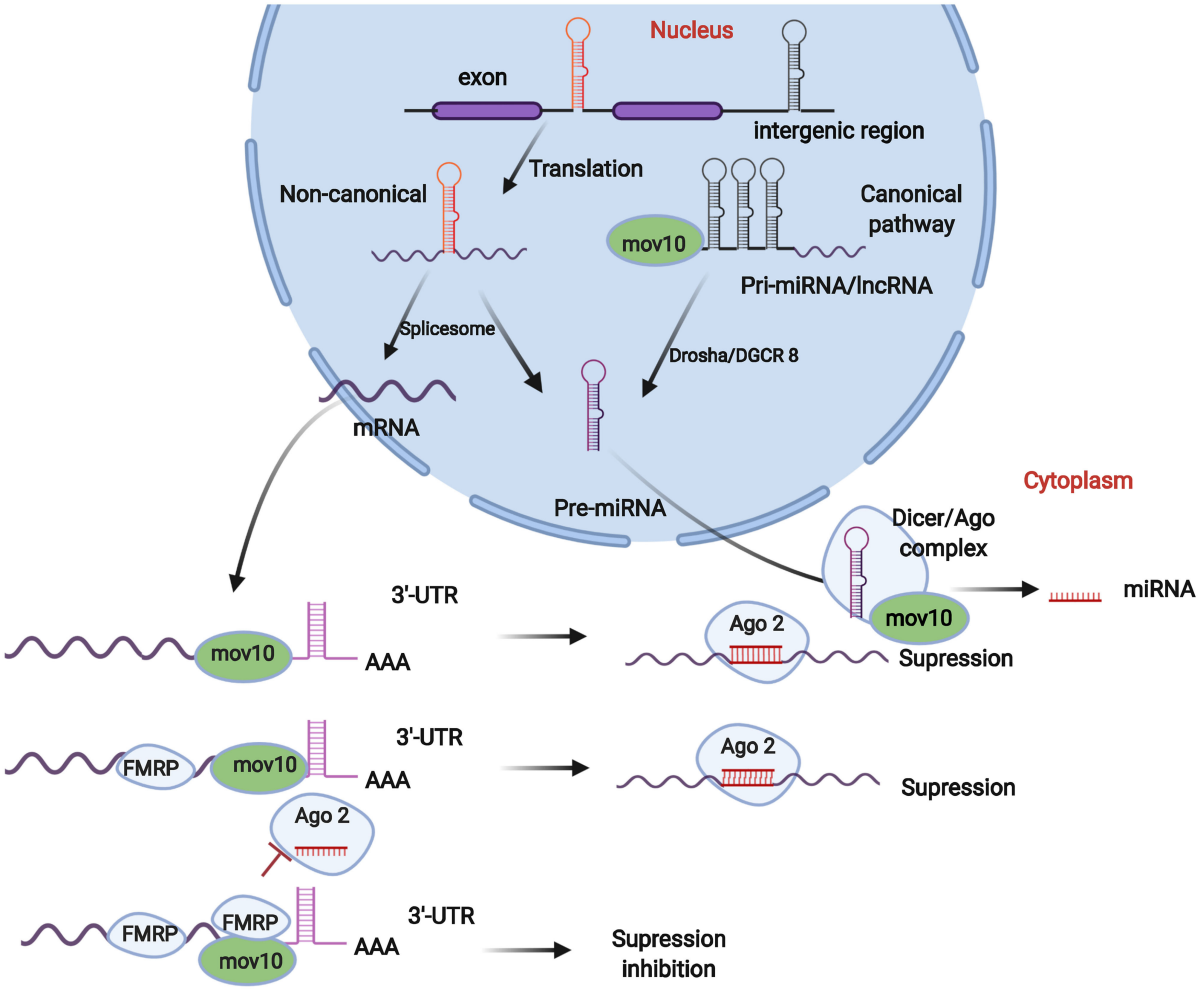
Roles of MOV10 in miRNA generation and mRNA stability. The MOV10 gene regulates the generation of miRNAs. Pre-miRNAs produced in the cell nucleus are generated from pri-miRNAs. The production of pri-miRNAs typically occurs in intergenic and intronic regions of the genome, which are then spliced into pre-miRNAs by a spliceosome (non-canonical pathway) or Drosha/DGCR8 enzymes (canonical pathway). In this process, MOV10 participates in the generation and cleavage of pri-miRNAs. Subsequently, miRNAs mature in the Dicer/Ago complex in cytoplasm. MOV10 is an important factor affecting the composition of the Dicer/Ago complex and plays crucial roles in miRNA maturation. MOV10 is also stored in the P body in cytoplasm and plays crucial roles in mRNA maturation, degradation, and stabilization, which contribute to maintaining the balance in miRNA, lncRNA, and mRNA pools in cells.

As mentioned above, MOV10 antiviral activity is expressed through the activation of different genes and pathways, of which miRNA pathway-mediated viral inhibition is important in restricting viral invasion. In the entire synthesis process of miRNAs, MOV10 not only functions in pri-miRNA production in the cell nucleus (68) but also has crucial roles in miRNA maturation and degradation (69). MOV10 also has crucial roles in mRNA maturation and degradation via exposure of miRNA recognition sites within mRNAs (Figure 2) (70).

The generation of pri-miRNAs and pre-miRNAs is the first step in the production of miRNAs, which is regulated by Drosha and DGCR8 enzymes and is also controlled by the MOV10 gene (71). Generally, the original pool is composed of pri-miRNAs and pre-mRNAs. Pre-miRNAs are typically produced in the cell nucleus by two important pathways. In the canonical pathway, pre-miRNAs are generated from exonic, intronic, or intergenic regions, followed by Drosha/DGCR8 processing to transform pri-miRNA transcripts into pre-miRNAs (72, 73). In the non-canonical pathway, pre-miRNAs are formed by splicing, debranching, and trimming of short introns without Drosha processing (74). In this process to form pre-miRNAs, MOV10 typically binds splicing factors, such as SRSF1 and DDX5 (68, 75), forming a splicing complex, which completes the pre-mRNA and pri-miRNA splicing process after binding intronic regions (Figure 2).

The pre-miRNAs generated from the canonical and non-canonical pathways are exported from the nucleus via Exportin 5 and then cleavage by Dicer occurs within the RISC loading complex (RLC) (76). Then, the miRNA/miRNA duplex unwinds via the Argonaute complex. In this process, MOV10 typically combines with Dicer, Ago, and TRBP (transactivation response element RNA-binding protein) forming the RISC complex and finally promoting miRNA maturation (76). Thus, MOV10 deficiency could cause an imbalance in the pools of miRNA, mRNA, and lncRNA (long non-code RNA) (Figure 2).

MOV10 is also important in the regulation of mRNA degradation and stability. A MOV10 protein, typically in the cytoplasm, combines with an Argonaute protein to form a complex that was recognized as an important component stored in a mRNA processing body (the P body) (60). In a previous study (60, 66), MOV10 inhibited retrotransposition by binding cell ribonucleoprotein particles (RNPs). MOV10 can bind high GC regions of the 3′-UTR within mRNAs, exposing miRNA recognition sites and guiding Ago proteins in promoting mRNA degradation (60). MOV10 can also increase mRNA stability and maturity via enriching the Fragile X mental retardation protein (FMRP) in miRNA-recognized sites (Figure 2) (66).

The virulence and infectivity of RNA viruses are influenced by MOV10-mediated changes in miRNA/mRNA. In the third stage of HIV infection, HIV infectivity is weakened because of miRNA expression in target cells (63). MOV10 also serves as a cofactor of HIV-1 Rev to facilitate Rev/RRE-dependent nuclear export of viral mRNAs (77). The same influence of MOV10 is apparent in the replication of human hepatitis delta virus (57). MOV10 can also weaken influenza infectivity and inhibit influenza A virus replication via binding RNP (65, 78).

## Conclusions

The antiviral activity of MOV10 during RNA virus infection is discussed in this review. Overall, the antiviral capacity of MOV10 is primarily reflected in two important mechanisms. First, MOV10 weakens viral virulence by binding the related protein, thereby activating IFN signal and cell autophagy pathways. Second, MOV10 functions as an RNA helicase during RNA virus infection. MOV10 regulates miRNA and mRNA generation, maturation, and degradation by miRNA pathways, which influence virus replication and packaging. The two mechanisms are typically coordinated in different virus infection stages. In addition, the number of studies is increasing that confirm MOV10 not only has important roles in infection by human viruses, such as HIV and human hepatitis delta virus, but also shows antiviral activity against animal RNA viruses. The antiviral ability of MOV10 and the related pathways during animal RNA virus infection are worthy of future attention. It is also can be used as an important tools for reduction of animal RNA viruses infection in future.

## Author Contributions

FS, XL, and YJ conceived the study and wrote the manuscript. All authors contributed to the article and approved the submitted version.

## Conflict of Interest

The authors declare that the research was conducted in the absence of any commercial or financial relationships that could be construed as a potential conflict of interest.
